# The impact of onset-to-cut time in surgery for stable acute type A aortic dissection—a single-centre retrospective cohort study

**DOI:** 10.1093/icvts/ivae130

**Published:** 2024-07-05

**Authors:** Leonard Pitts, Markus Kofler, Matteo Montagner, Roland Heck, Stephan Dominik Kurz, Alexandru Claudiu Paun, Volkmar Falk, Jörg Kempfert

**Affiliations:** Deutsches Herzzentrum der Charité (DHZC), Department of Cardiothoracic and Vascular Surgery, Augustenburger Platz 1, Berlin 13353, Germany; Charité – Universitätsmedizin Berlin, corporate member of Freie Universität Berlin and Humboldt-Universität zu Berlin, Charitéplatz 1, Berlin 10117, Germany; Deutsches Herzzentrum der Charité (DHZC), Department of Cardiothoracic and Vascular Surgery, Augustenburger Platz 1, Berlin 13353, Germany; Charité – Universitätsmedizin Berlin, corporate member of Freie Universität Berlin and Humboldt-Universität zu Berlin, Charitéplatz 1, Berlin 10117, Germany; DZHK (German Centre for Cardiovascular Research), partner site Berlin, Germany; Deutsches Herzzentrum der Charité (DHZC), Department of Cardiothoracic and Vascular Surgery, Augustenburger Platz 1, Berlin 13353, Germany; Charité – Universitätsmedizin Berlin, corporate member of Freie Universität Berlin and Humboldt-Universität zu Berlin, Charitéplatz 1, Berlin 10117, Germany; Deutsches Herzzentrum der Charité (DHZC), Department of Cardiothoracic and Vascular Surgery, Augustenburger Platz 1, Berlin 13353, Germany; Charité – Universitätsmedizin Berlin, corporate member of Freie Universität Berlin and Humboldt-Universität zu Berlin, Charitéplatz 1, Berlin 10117, Germany; Deutsches Herzzentrum der Charité (DHZC), Department of Cardiothoracic and Vascular Surgery, Augustenburger Platz 1, Berlin 13353, Germany; Charité – Universitätsmedizin Berlin, corporate member of Freie Universität Berlin and Humboldt-Universität zu Berlin, Charitéplatz 1, Berlin 10117, Germany; Deutsches Herzzentrum der Charité (DHZC), Department of Cardiothoracic and Vascular Surgery, Augustenburger Platz 1, Berlin 13353, Germany; Charité – Universitätsmedizin Berlin, corporate member of Freie Universität Berlin and Humboldt-Universität zu Berlin, Charitéplatz 1, Berlin 10117, Germany; Deutsches Herzzentrum der Charité (DHZC), Department of Cardiothoracic and Vascular Surgery, Augustenburger Platz 1, Berlin 13353, Germany; Charité – Universitätsmedizin Berlin, corporate member of Freie Universität Berlin and Humboldt-Universität zu Berlin, Charitéplatz 1, Berlin 10117, Germany; DZHK (German Centre for Cardiovascular Research), partner site Berlin, Germany; Department of Health Sciences and Technology, Translational Cardiovascular Technologies, Institute of Translational Medicine, Swiss Federal Institute of Technology (ETH), Zurich, Switzerland; Deutsches Herzzentrum der Charité (DHZC), Department of Cardiothoracic and Vascular Surgery, Augustenburger Platz 1, Berlin 13353, Germany; Charité – Universitätsmedizin Berlin, corporate member of Freie Universität Berlin and Humboldt-Universität zu Berlin, Charitéplatz 1, Berlin 10117, Germany; DZHK (German Centre for Cardiovascular Research), partner site Berlin, Germany

**Keywords:** Acute type A aortic dissection, Malperfusion, Onset-to-cut time, Surgery, Referral, Aorta

## Abstract

**OBJECTIVES:**

The goal of this study was to investigate the impact of onset-to-cut time on mortality in patients undergoing surgery for stable acute type A aortic dissection.

**METHODS:**

Patients who underwent surgery for acute type A aortic dissection between January 2006 and December 2021 and available onset-to-cut times were included. Patients with unstable aortic dissection (preoperative shock, intubation, resuscitation, coma, pericardial tamponade and local/systemic malperfusion syndromes) were excluded. After descriptive analysis, a multivariable binary logistic regression for 30-day mortality was performed. A receiver operating characteristic curve for onset-to-cut time and 30-day mortality was calculated. Restricted cubic splines were designed to investigate the association between onset-to-cut time and survival.

**RESULTS:**

The final cohort comprised 362 patients. The median onset-to-cut time was 543 (376–1155) min. The 30-day mortality was 9%. Only previous myocardial infarction (*P* = 0.018) and prolonged cardiopulmonary bypass time (*P* < 0.001) were identified as independent risk factors for 30-day mortality. The corresponding area under the receiver operating characteristic curve showed a value of 0.49. Restricted cubic splines did not indicate an association between onset-to-cut time and survival (*P* = 0.316).

**CONCLUSIONS:**

Onset-to-cut time in the setting of stable acute type A aortic dissection does not seem to be a valid predictor of 30-day mortality in patients undergoing surgery and stayed stable during the preoperative course.

**Figure:**
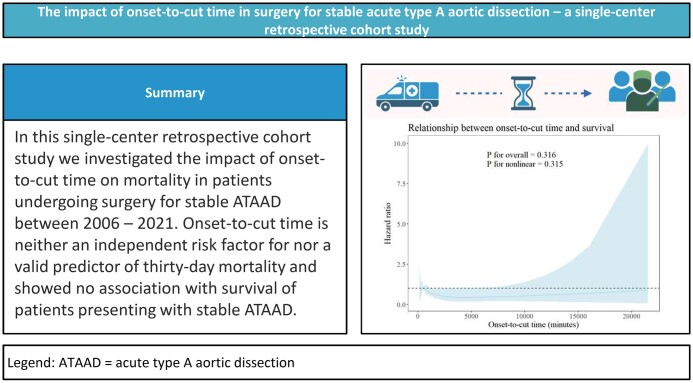

## INTRODUCTION

Acute type A aortic dissection (ATAAD) is associated with high morbidity and mortality [1, 2]. Previously published data suggest that in the first 24–48 h after symptom onset, mortality is ∼1–2% per h without surgical treatment [3]. These data led to the recommendation to perform an immediate operation irrespective of the time of day and of the availability of specialized teams, often resulting in hemiarch replacement [4, 5]. Lately, a tailored approach to the timing of the operation for ATAAD that considers patient-, disease- and service-related factors has been gaining more interest [6–9].

Patients presenting with unstable ATAAD due to malperfusion, stroke, pericardial tamponade, preoperative shock and/or preoperative resuscitation are at a higher risk than those with stable ATAAD [10–14]. These factors not only define the surgical outcome per se but also have an impact on the timing and the event rate until surgery for unstable ATAAD. In stable ATAAD, the impact of timing for surgery is less clear. The goal of our study was to investigate the impact of onset-to-cut time on mortality in patients undergoing surgery for stable acute type A aortic dissection.

## PATIENTS AND METHODS

### Ethics approval

Approval was granted by the institutional review board for this study on 6 November 2020 (No. EA2/096/20). It complies with the Declaration of Helsinki.

### Patient population

The study design followed the STROBE (STrengthening the Reporting of OBservational studies in Epidemiology) statement. All patients who underwent surgical repair for ATAAD at our institution between January 2006 and December 2021 were consecutively collected and included in this single-centre retrospective cohort study. Patients with iatrogenic ATAAD and patients with subacute/chronic aortic dissection (onset of dissection 14 days or more) were excluded. Onset-to-cut time, defined as the time from the first onset of symptoms until the start of surgery, was evaluated for every patient. To this end, emergency physician protocols and all available medical reports were investigated to determine the time of symptom onset. Patients with unidentifiable onset-to-cut times were then excluded. Finally, all patients presenting with unstable ATAAD were excluded.

### Definition of stable and unstable acute type A aortic dissection

Each patient was investigated for factors constituting unstable ATAAD. These were defined as follows: preoperative shock, preoperative intubation, preoperative resuscitation, preoperative coma, pericardial tamponade and local organ malperfusion syndrome. Pericardial effusion alone was no criterion constituting unstable ATAAD. The diagnosis of organ malperfusion (coronary, cerebral, spinal, visceral, renal and peripheral) was based mainly on clinical and laboratory findings. Where available, computed tomography (CT) data were also considered. Criteria for organ malperfusion were recently described and are summarized in Supplementary Material, Table S1 [11]. If any of the preceding factors applied to a patient, ATAAD was defined as unstable, and the patient was excluded. Patients were also excluded if symptoms occurred only temporarily prior to the operation or had resolved by the time of admission. Stable ATAAD was defined as an awake and haemodynamically stable patient with no clinical and/or radiologic signs of local or systemic malperfusion until the start of the operation.

**Table 1:** Pre- and intraoperative variables.

Preoperative variables	Stable ATAAD	Intraoperative variables	Stable ATAAD
*N* (%)/median (IQR)	(*n* = 362)	*N* (%)/median (IQR)	(*n* = 362)
Onset-to-cut time (min)	543 (376–1155)	Time period (>2014)	192 (53)
Gender (female)	127 (35)	Nighttime surgery	192 (53)
Age (years)	60 (52–70)	CPB time (min)	210 (168–261)
BMI (kg/m²)	26 (24–29)	Cross-clamp time (min)	100 (81–125)
PAD	15 (4)	Circulatory arrest time (min)	35 (25–44)
Diabetes mellitus	34 (9)	Core temperature (°C)	26 (18–28)
COPD	25 (7)	Antegrade unilateral cerebral perfusion	127 (35)
CKD	42 (12)	Antegrade bilateral cerebral perfusion	76 (21)
CAD	36 (10)	Retrograde cerebral perfusion	109 (30)
Previous MI	14 (4)	Femoral arterial cannulation	129 (36)
Previous stroke	18 (5)	Axillary arterial cannulation	203 (56)
Previous aortic pathology	51 (14)	Aortic root replacement	83 (23)
Previous cardiac surgery	14 (4)	Aortic arch replacement (FET)	33 (9)
LV dysfunction (LVEF < 50%)	21 (6)	Concomitant coronary bypass	7 (2)

ATAAD: acute type A aortic dissection; BMI: body mass index; CAD: coronary artery disease; CKD: chronic kidney disease; COPD: chronic obstructive pulmonary disease; CPB: cardiopulmonary bypass; FET: frozen elephant trunk; IQR: interquartile range (25th–75th percentile); LV: left ventricular; LVEF: left ventricular ejection fraction; MI: myocardial infarction; PAD: peripheral artery disease.

### Preoperative management and surgical procedure

The patients were referred from different hospitals in our city and its surrounding region, which include more than 6 million inhabitants. Since 2015, our aortic hotline contributes to a standardized rescue chain for peripheral hospitals and aids in patient referral to our clinic. The rescue chain included either transfer on the ground or by air. Patients were routinely screened during the preoperative course for clinical signs of unstable ATAAD, complemented by the findings of a CT scan, which was performed at the referring hospital and sent to us for detailed assessment of ATAAD and surgical planning. Patients were treated with demand-oriented opioid analgesics and received intravenous antihypertensive medication to control blood pressure measured via the left radial artery. An additional left femoral artery catheter was placed to enable blood pressure tracing of the downstream aorta during the operation. All patients underwent surgery immediately after admission regardless of the time and day as per our ATAAD standard operating procedure. An operation, of which >50% took place between 6 p.m. and 6 a.m., was defined as a nighttime operation. The right axillary artery and the right femoral artery were the preferred arterial cannulation sites. Cardiopulmonary bypass and systemic cooling were initiated. Depending on the changing concept of cerebral protection over the study period, the level of hypothermia was adjusted to the selected route of cerebral perfusion [15]. Retrograde cerebral perfusion with the patient under deep hypothermia was performed until 2014, whereas antegrade cerebral perfusion under moderate hypothermia was performed mainly since 2015 [16]. After inspection of the entry site, the ascending aorta was resected with or without the aortic arch. Total arch replacement using the frozen elephant trunk technique was performed in case of an aortic arch aneurysm or aortic arch entry tear. Whenever possible, the aortic root was preserved and reconstructed. Valve-sparing root replacement was restricted to very selected cases. If reconstruction of the aortic root proved impossible, composite replacement with a valved graft conduit and reimplantation of the coronary arteries were performed.

### Definition of outcome variables

Thirty-day mortality and 1-year survival were the primary end points. Survival data of the study cohort were collected during postoperative appointments in our aortic outpatient clinic, by our study centre and via telephone calls. The follow-up was 100% complete for 30-day mortality and 98% complete for 1-year survival [17]. Only 8 patients (2%) were lost to follow-up during the first postoperative year after discharge. The median follow-up time was 1074 (488–2030) days. Follow-up was closed in March 2024.

Single secondary end points were defined as follows: revision for malperfusion included all surgical and interventional procedures to treat postoperative malperfusion syndromes (percutaneous coronary intervention, stenting of branch vessels, visceral surgery, fasciotomy). Re-thoracotomy was performed for postoperative bleeding complications or pericardial tamponade. The diagnosis of postoperative stroke required the confirmation of cerebral ischaemia or haemorrhage by cerebral CT.

### Statistical analysis

Categorical data are presented as absolute numbers with corresponding percentages. Continuous variables were tested for normal distribution using the Shapiro–Wilk test and were visualized using histograms. All continuous variables exhibited a non-normal distribution. Consequently, they were presented as medians with corresponding interquartile ranges (25th–75th percentile). A multivariable binary logistic regression analysis was performed to identify independent risk factors for 30-day mortality. Cox analysis was waived because a proportional hazard assumption was not given. All variables included in Table 1 (pre- and intraoperative variables) were considered in the initial logistic regression model. The study period was also considered as a binary covariate due to the different operative and preoperative management protocols during the time periods. After checking for relevant multicollinearity, the variables for the regression model were selected using the backward selection technique based on the Akaike information criterion. All selected variables were then used for the multivariable binary logistic regression and are shown in Table 3. Complementary univariable binary logistic regression was performed. A receiver operating curve was designed to measure the prediction accuracy of onset-to-cut time for 30-day mortality. The corresponding area under the curve was defined as acceptable with a value > 0.70. Restricted cubic splines were used to investigate the association between onset-to-cut time and survival. A Kaplan–Meier curve including patients at risk was used to visualize 1-year survival in patients undergoing surgery for stable ATAAD. All *P*-values are two-sided. The α-level was defined as 0.05. Statistical analyses were performed using R (The R Foundation for Statistical Computing, Vienna, Austria) version 4.2.3.

**Table 2:** Postoperative variables.

Postoperative variables	Stable ATAAD
*N* (%)/median (IQR)	(*n* = 362)
ICU treatment (days)	5 (2–13)
Ventilation time (days)	2 (1–7)
Tracheotomy	47 (13)
Postoperative ECLS	4 (1)
Postoperative dialysis	25 (7)
Revision for malperfusion	27 (7)
Revision for bleeding	68 (19)
CT-confirmed stroke	43 (12)
Thirty-day mortality	33 (9)

ATAAD: acute type A aortic dissection; CT: computed tomography; ECLS: extracorporeal life support; ICU: intensive care unit; IQR: interquartile range (25th–75th percentile).

**Table 3:** Results of binary logistic regression for 30-day mortality in stable ATAAD.

	Univariable binary logistic regression			Multivariable binary logistic regression		
Variables	OR	95% CI	*P*-value	OR	95% CI	*P*-value
Onset-to-cut time	1.000	0.999–1.000	0.314	1.000	0.999–1.000	0.153
Time period (< 2014)	0.753	0.360–1.561	0.446	0.566	0.226–1.432	0.223
Gender (female)	1.514	0.715–3.147	0.269	1.904	0.834–4.333	0.122
Diabetes mellitus	2.698	0.938–6.794	0.046	2.564	0.765–7.470	0.099
Previous MI	6.605	1.918–20.575	0.001	6.266	1.400–25.612	0.018
CPB time	1.001	1.005–1.014	< 0.001	1.011	1.010–1.012	< 0.001
Core temperature	0.986	0.932–1.043	0.633	1.066	0.934–1.153	0.112

CI: confidence interval; CPB: cardiopulmonary bypass; MI: myocardial infarction; OR: odds ratio.

## RESULTS

### Pre- and intraoperative variables

**Figure 1:**
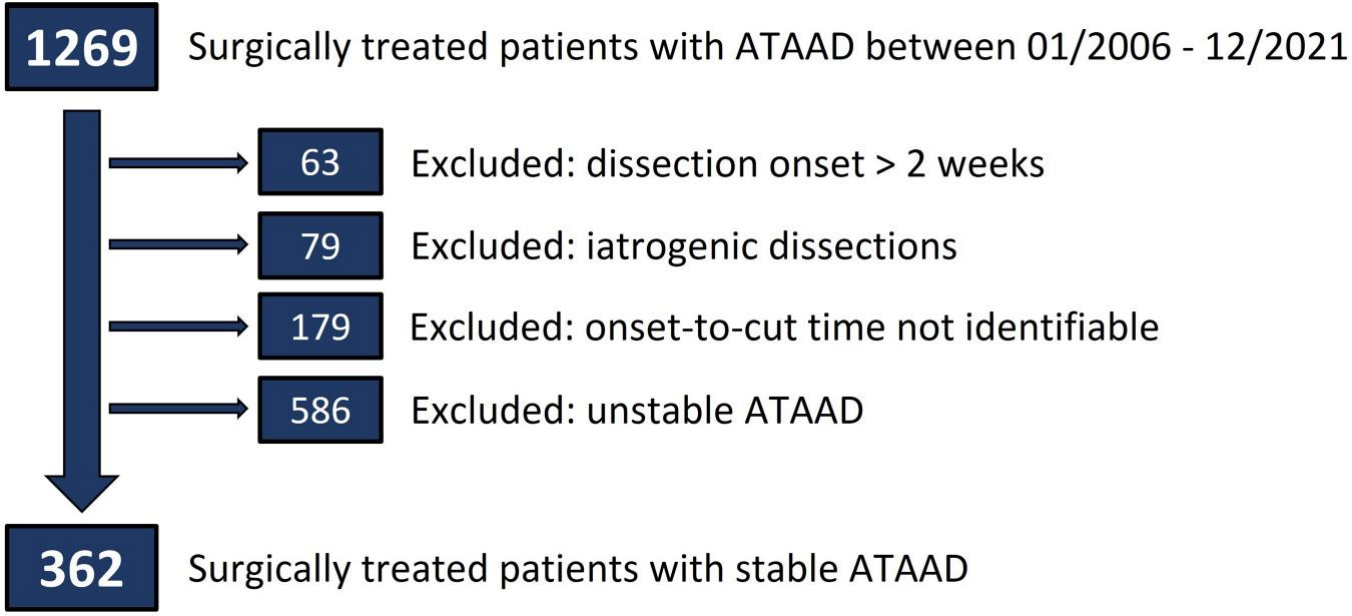
Flow chart for group formation and patient selection.

After excluding patients with a dissection onset > 2 weeks (*n* = 63), patients with iatrogenic ATAAD (*n* = 79) and patients with an unidentifiable onset-to-cut time (*n* = 179), patients with unstable ATAAD (*n* = 586) were also excluded. The final cohort comprised 362 patients. The corresponding flow chart is shown in Fig. 1. Pre- and intraoperative variables are shown in Table 1. The onset-to-cut time was 543 (376–1155) min or 9 (6–19) h. Patients were 60 (52–70) years of age and one-third were female. A prior aortic pathology in the form of thoracic and/or abdominal aortic aneurysms (with or without previous intervention) was observed in 51 (14%) patients. Previous stroke (5%), previous myocardial infarction (4%) and previous cardiac surgery (4%) were observed infrequently. The distribution between the study periods (> 2014 vs < 2014) was balanced, including 192 (53%) patients in the study period > 2014, which also constituted the number of cases needing nighttime surgery (53%). The average core temperature was 26 (18–28)°C. Axillary artery cannulation was performed in 203 (56%) patients, followed by femoral cannulation in 129 (36%) patients. Retrograde cerebral perfusion was performed in 109 (30%) patients, whereas unilateral and bilateral antegrade cerebral perfusion was performed in 127 (35%) and 76 (21%) patients, respectively. Aortic root replacement was performed in 83 (23%) patients and total arch replacement using a frozen elephant trunk, in 33 (9%) patients.

### Postoperative variables

The postoperative variables are shown in Table 2. The median time on the intensive care unit was 5 (2–13) days and the ventilation time was 2 (1–7) days; 47 (13%) patients underwent tracheotomy in the further course. Postoperative extracorporeal life support, including intra-aortic balloon pump or veno-arterial extracorporeal membrane oxygenation, was necessary in 4 (1%) patients. Revision for malperfusion was performed in 27 (7%) patients, and 68 (19%) patients underwent re-thoracotomy for bleeding. Postoperative CT-confirmed stroke was detected in 43 (12%) patients. Thirty-day mortality was 9% for the cohort.

### Influence of onset-to-cut time

**Figure 2:**
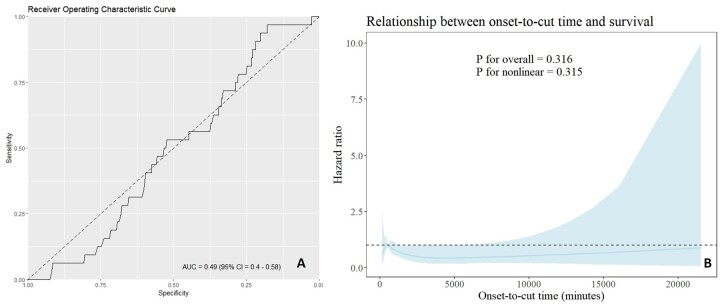
(**A**) Receiver operating curve for onset-to-cut time and 30-day mortality in stable acute type A aortic dissection. (**B**) Restricted cubic splines showing the relationship between onset-to-cut time and survival in stable acute type A aortic dissection. ATAAD: acute type A aortic dissection; CI: confidence interval.

**Figure 3:**
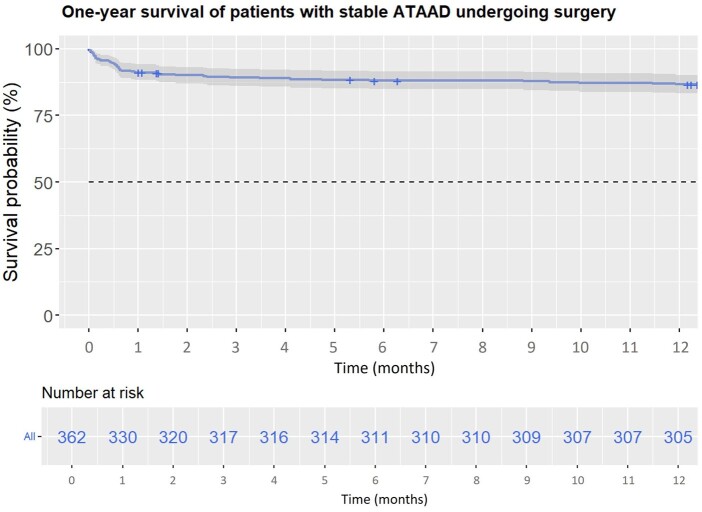
Kaplan–Meier curve showing 1-year survival for patients undergoing surgery for stable acute type A aortic dissection. ATAAD: acute type A aortic dissection.

The results of the uni- and multivariable binary logistic regressions are shown in Table 3. Because the variable ‘onset-to-cut time’ was not selected during the variable selection process using the Akaike information criterion for the multivariable logistic regression, it was forced into the regression model. Neither the univariable logistic regression (*P* = 0.314) nor the multivariable logistic regression (*P* = 0.153) identified onset-to-cut time as an independent risk factor for 30-day mortality in stable ATAAD. Instead, previous myocardial infarction (*P* = 0.018) and prolonged cardiopulmonary bypass time (*P* < 0.001) were identified as independent risk factors for 30-day mortality in stable ATAAD. The receiver operating curve for onset-to-cut time and 30-day mortality is shown in Fig. 2A. The corresponding area under the curve showed a value of 0.49, which was non-sufficient. Restricted cubic splines are shown in Fig. 2B, indicating no relevant association between onset-to-cut time and survival (*P* for overall = 0.316, *P* for nonlinear = 0.315). One-year survival is depicted in Fig. 3 as a Kaplan–Meier curve, showing 8 (2%) patients lost to follow-up after discharge during the first year.

## DISCUSSION

In this unicentric retrospective study, we evaluated the impact of onset-to-cut time on perioperative mortality in the surgical treatment of stable ATAAD. The preoperative course and the influence of onset-to-cut time have been the subjects of much debate, based largely on a widely cited untreated mortality rate of 1–2% per hour [3, 6]. Interestingly, a recent study based on data from the International Registry of Acute Aortic Dissection revealed that non-operated patients presenting with ATAAD exhibited a mortality rate of only 0.5% per h in the first 48 h [18]. This rate went down to 0.09% per h for those undergoing surgery or designated to undergo surgery. The median onset-to-cut time was 6 h (4.0–15.0). Furthermore, 1% of patients scheduled to undergo surgical treatment died before an operation took place. Compared with those who underwent an operation, these patients were older and more likely to present with unstable ATAAD. These data provide an important and urgently needed update about the spontaneous course of ATAAD and about patients receiving only medical treatment. However, interpretation of these data is limited because there was no stratification for unstable and stable ATAAD. Instead, conclusions were drawn for the cohort as a whole. There is general agreement to perform immediate surgery in case of unstable ATAAD to resolve malperfusion or deal with cardiac tamponade.

A recent study investigating the impact of preoperative malperfusion and onset-to-cut time on the surgical outcome showed reduced survival in case of delayed surgery for unstable ATAAD, driven mainly by the presence of preoperative malperfusion [19]. Based on this observation, the authors concluded that the operation should be performed preferably within 5 h after symptom onset to improve the long-term outcome (*P* = 0.03). In contrast, other studies found an inverse relationship between onset-to-cut time and perioperative mortality [18]. Gasser and colleagues identified patients who underwent surgery within 4 h as an exclusive high-risk cohort with significantly higher rates of preoperative malperfusion [6]. This result might be due to the natural course of the disease resulting in an over-representation of critically ill and symptomatic patients with unstable ATAAD, leading to a significantly shorter onset-to-cut time [20]. Multiple factors might contribute to this scenario, including faster referral to a hospital after onset of symptoms and, in turn, a more rapid diagnosis, which makes inferences to the onset-to-cut time complex [21]. Besides, all these findings relate to patients with unstable ATAAD; no conclusion can be drawn from this about patients with stable ATAAD. Our data suggest that the impact of onset-to-cut time might be marginal in case of stable ATAAD. The threat of secondary aortic rupture–which speaks in favour of early rather than late intervention–exists, though the risk might be lower than generally expected: of 5131 (91.4%) patients who were scheduled to undergo or actually underwent surgery registered in the International Registry of Acute Aortic Dissection, only 51 (1%) patients who were deemed candidates for surgery died while awaiting surgery [18]. The cause of death in these patients was mainly malperfusion; secondary aortic rupture was rare and occurred in 13 (0.25%) patients. Considering these circumstances, the time to surgery might be less important in stable ATAAD than expected. This may be of great relevance for patients with stable ATAAD who present in a haemodynamically stable condition but require technically challenging operations like total arch replacement with a frozen elephant trunk or valve-sparing aortic root replacement [22]. These operations should preferably not be performed by the on-call team but rather by a specialized aortic team including an aortic surgeon. This concept is supported by recent findings from Harky *et al.*, who showed that the impact of a dedicated aortic team may be greater than the timing of the operation [23]. However, we must keep in mind that preoperative malperfusion in ATAAD is a dynamic process and can occur temporarily or even develop in the further course after an asymptomatic beginning. It might be the Achilles’ heel in operative management of ATAAD to weigh out the benefits of immediate surgery in terms of solely hemiarch replacement against the benefits of surgery by an experienced aortic surgeon. Therefore, it is of upmost importance to re-evaluate patients precisely and react immediately if stable ATAAD progresses to unstable ATAAD.

### Limitations

This study is limited by its unicentric and retrospective nature. Furthermore, it lacks data on patients dying before hospitalization or designated to undergo surgery, which could have influenced the results. However, given the fact that data for such patients are not available and onset-to-cut time is not definable, it would not have been feasible to consider them in the analysis. Moreover, we excluded all patients with an unidentifiable onset-to-cut time from the final analysis, which might result in certain selection bias and make conclusions invalid for patients who re-present with asymptomatic ATAAD. Though maximum effort was made to investigate the actual time point of symptom onset, certain bias cannot be ruled out due to inaccuracy in terms of emergency physician protocols and patient anamnesis.

## CONCLUSIONS

Onset-to-cut time is not an independent risk factor for 30-day mortality in patients undergoing surgery for stable ATAAD and staying stable during the preoperative course. It is not a valid predictor of 30-day mortality, bearing in mind that these conclusions are only applicable to this specific patient cohort. The decision for immediate surgery in case of stable ATAAD should be balanced against the benefits of surgery by an experienced aortic team. The operation should still be carried out quickly, considering the dynamic nature of ATAAD, and no compromises should be made if stable ATAAD becomes unstable.

## Supplementary Material

ivae130_Supplementary_Data

## Data Availability

The data underlying this article are available in the article and in its supplementary online material. Further requests for data can be made to the corresponding author.

## Reviewer information

**Leonard Pitts:** Conceptualization; Data curation; Formal analysis; Investigation; Methodology; Project administration; Resources; Software; Validation; Visualization; Writing—original draft; Writing—review & editing. **Markus Kofler:** Conceptualization; Methodology; Project administration; Supervision; Writing—review & editing. **Matteo Montagner:** Data curation; Supervision; Writing—review & editing. **Roland Heck:** Data curation; Supervision; Writing—review & editing. **Stephan Dominik Kurz:** Data curation; Supervision; Writing—review & editing. **Alexandru Claudiu Paun:** Data curation; Supervision; Writing—review & editing. **Volkmar Falk:** Resources; Writing—review & editing. **Jörg Kempfert:** Conceptualization; Investigation; Methodology; Project administration; Supervision; Validation; Writing—review & editing.

## ABBREVIATIONS

ATAAD: Acute type A aortic dissection
CT: Computed tomography

## References

[1] Pitts L , Montagner M, Kofler M, Van Praet KM, Heck R, Buz S et al State of the art review: surgical treatment of acute type A aortic dissection. Surg Technol Int 2021;38:279–88. 33823055

[2] Kurz SD , Falk V, Kempfert J, Gieb M, Ruschinski TM, Kukucka M et al Insight into the incidence of acute aortic dissection in the German region of Berlin and Brandenburg. Int J Cardiol 2017;241:326–9. 28499667 10.1016/j.ijcard.2017.05.024

[3] Hirst AE Jr , Johns VJ Jr, Kime SW Jr. Dissecting aneurysm of the aorta: a review of 505 cases. Medicine (Baltimore) 1958;37:217–79. 13577293 10.1097/00005792-195809000-00003

[4] Montagner M , Kofler M, Seeber F, Pitts L, Starck C, Sundermann SH et al The arch remodelling stent for DeBakey I acute aortic dissection: experience with 100 implantations. Eur J Cardiothorac Surg 2022;62:ezac384. 10.1093/ejcts/ezac384 35809065

[5] Pitts L , Moon MC, Luehr M, Kofler M, Montagner M, Sündermann S et al The Ascyrus medical dissection stent: a one-fits-all strategy for the treatment of acute type A aortic dissection? J Clin Med 2024;13:2593. 38731123 10.3390/jcm13092593 PMC11084383

[6] Gasser S , Stastny L, Kofler M, Krapf C, Bonaros N, Grimm M et al Rapid response in type A aortic dissection: is there a aecisive time interval for surgical repair? Thorac Cardiovasc Surg 2021;69(1):49–56. 32114688 10.1055/s-0039-1700967

[7] Gasser S , Stastny L, Kofler M, Zujs V, Krapf C, Semsroth S et al Surgery out of office hours for type A aortic dissection: does night-time and weekend surgery worsen outcome? Interact CardioVasc Thorac Surg 2020;31:806–12. 33001169 10.1093/icvts/ivaa190

[8] Pitts L , Heck R, Montagner M, Penkalla A, Kofler M, Falk V et al Case report: successful endovascular treatment of acute type A aortic dissection. Front Cardiovasc Med 2023;10. 10.3389/fcvm.2023.1299192. PMC10687577 38034371

[9] Sabe AA , Percy ED, Kaneko T, Plichta RP, Hughes GC. When to consider deferral of surgery in acute type A aortic dissection: a review. Ann Thorac Surg 2021;111:1754–62. 32882193 10.1016/j.athoracsur.2020.08.002 PMC7457910

[10] Dumfarth J , Peterss S, Luehr M, Etz CD, Schachner T, Kofler M et al Acute type A dissection in octogenarians: does emergency surgery impact in-hospital outcome or long-term survival? Eur J Cardiothorac Surg 2017;51:472–7. 28364444 10.1093/ejcts/ezw387

[11] Pitts L , Kofler M, Montagner M, Heck R, Kurz SD, Buz S et al The impact of malperfusion patterns in elderly patients undergoing surgery for acute type A aortic dissection. Eur J Cardiothorac Surg 2023;64(4):ezad288. 10.1093/ejcts/ezad288 37589652

[12] Dumfarth J , Kofler M, Stastny L, Gasser S, Plaikner M, Semsroth S et al Immediate surgery in acute type A dissection and neurologic dysfunction: fighting the inevitable? Ann Thorac Surg 2020;110(1):5–12. 32114042 10.1016/j.athoracsur.2020.01.026

[13] Dumfarth J , Kofler M, Stastny L, Plaikner M, Krapf C, Semsroth S et al Stroke after emergent surgery for acute type A aortic dissection: predictors, outcome and neurological recovery. Eur J Cardiothorac Surg 2018;53:1013–20. 29360972 10.1093/ejcts/ezx465

[14] Dumfarth J , Peterss S, Kofler M, Plaikner M, Ziganshin BA, Schachner T et al In DeBakey type I aortic dissection, bovine aortic arch is associated with arch tears and stroke. Ann Thorac Surg 2017;104:2001–8. 28811002 10.1016/j.athoracsur.2017.05.026

[15] Pitts L , Kofler M, Montagner M, Heck R, Iske J, Buz S et al Cerebral protection strategies and stroke in surgery for acute type A aortic dissection. J Clin Med 2023;12:2271. 36983272 10.3390/jcm12062271 PMC10056182

[16] Montagner M , Kofler M, Pitts L, Heck R, Buz S, Kurz S et al Matched comparison of 3 cerebral perfusion strategies in open zone-0 anastomosis for acute type A aortic dissection. Eur J Cardiothorac Surg 2022;62(5):ezac214. 10.1093/ejcts/ezac214 35396839

[17] Thuijs DJFM , Hickey GL, Osnabrugge RLJ. Statistical primer: basics of survival analysis for the cardiothoracic surgeon. Interact Cardiovasc Thorac Surg 2018;27:1–4. 29800119 10.1093/icvts/ivy010

[18] Harris KM , Nienaber CA, Peterson MD, Woznicki EM, Braverman AC, Trimarchi S et al Early mortality in type A acute aortic dissection: insights from the International Registry of Acute Aortic Dissection. JAMA Cardiol 2022;7:1009–15. 36001309 10.1001/jamacardio.2022.2718 PMC9403853

[19] Nakai C , Izumi S, Haraguchi T, Henmi S, Nakayama S, Mikami T et al Impact of time from symptom onset to operation on outcome of repair of acute type A aortic dissection with malperfusion. J Thorac Cardiovasc Surg 2023;165:984–91.e1. 33941373 10.1016/j.jtcvs.2021.03.102

[20] Dimagli A , Angelini GD. “Time is aorta?”: timeliness of surgical repair in type A aortic dissection. J Card Surg 2022;37:1661–3. 35340069 10.1111/jocs.16412 PMC9314949

[21] Zaschke L , Habazettl H, Thurau J, Matschilles C, Göhlich A, Montagner M et al Acute type A aortic dissection: Aortic Dissection Detection Risk Score in emergency care—surgical delay because of initial misdiagnosis. Eur Heart J Acute Cardiovasc Care 2020;9:S40–7. 32223297 10.1177/2048872620914931

[22] Pitts L , Van Praet KM, Montagner M, Kofler M, Falk V, Kempfert J. David procedure as valve-sparing root replacement. Surg Technol Int 2022;41. 10.52198/22.STI.41.CV1593 35623037

[23] Harky A , Mason S, Othman A, Shaw M, Nawaytou O, Harrington D et al Outcomes of acute type A aortic dissection repair: daytime versus nighttime. JTCVS Open 2021;7:12–20. 36003743 10.1016/j.xjon.2021.04.017 PMC9390141

