# Supplementary material for: The impact of onset-to-cut time in surgery for stable acute type A aortic dissection—a single-centre retrospective cohort study
Source: Interdiscip Cardiovasc Thorac Surg. 2024 Jul 5;39(1):ivae130. doi: 10.1093/icvts/ivae130 (PMC11272170; doi:10.1093/icvts/ivae130)
Supplement: ivae130_Supplementary_Data [file ivae130_supplementary_data.docx]

**Supplemental material**

**Table 1) Definition of local malperfusion**

| **Local malperfusion** | **Definition** |
| --- | --- |
| Coronary | ECG-based signs of ischaemia, elevation of cardiac enzymes, new wall motion abnormalities, visualisation in coronary angiography |
| Cerebral | Focal neurological deficits, anisocoria, preoperative coma, CT-based signs of cerebral ischaemia (infarction, oedema) |
| Spinal | Paraplegia |
| Visceral | Acute abdomen, bloody defecation, elevation of liver enzymes and lactate, CT-based signs of visceral ischaemia (infarction, vascular obliteration) |
| Renal | New anuria, retention of creatinine and urea, CT-based signs of renal ischaemia (infarction, vascular obliteration) |
| Peripheral | Pulse absence in combination with pallor and/or coldness and/or paralysis, elevation of creatine kinase, CT-based signs of peripheral ischaemia (vascular obliteration) |
| CT = computed tomography, ECG = electrocardiography | |
